# Automated Identification of Nursing Diagnoses and Interventions From Nursing Records Using a Retrieval-Augmented Large Language Model Approach: Quantitative Study

**DOI:** 10.2196/89850

**Published:** 2026-04-29

**Authors:** Minye Li, Dianjie Chen, Qun Xiao, Zhengji He, Yanyan Zhang, Jinlan Zhong, Yiwei Luo, Hui Ma

**Affiliations:** 1 Department of Nursing, Chinese People's Liberation Army General Hospital, 28 Fuxing Road, Haidian District, Beijing, Beijing, China, 86 13681197727; 2 Department of Infectious Diseases, 5th Medical Center, Chinese People's Liberation Army General Hospital, Beijing, Beijing, China; 3 School of Nursing, Southern Medical University, Guangzhou, Guangdong, China; 4 Department of Respiratory and Critical Care Medicine, 1st Medical Center, Chinese People's Liberation Army General Hospital, Beijing, Beijing, China

**Keywords:** large language model, LLM, retrieval-augmented generation, RAG, nursing terminology, Clinical Care Classification, nursing records, nursing informatics

## Abstract

**Background:**

Electronic health records (EHRs) have been widely adopted, but most nursing records remain in unstructured free-text format, which limits the secondary use of nursing data. Standardized terminologies improve semantic interoperability; however, manual annotation is labor intensive and yields inconsistent results. Advances in large language models (LLMs) and retrieval-augmented generation (RAG) have created new possibilities for automating the mapping of nursing records to standardized terminologies, thereby enhancing the utility of nursing data.

**Objective:**

This study aimed to develop and evaluate Clinical Care Classification nursing terminology with retrieval-augmented mapping (CNTRAM), a 2-stage RAG framework incorporating an LLM, for the automated mapping of nursing diagnoses and interventions from free-text intensive care unit nursing records to standardized Clinical Care Classification (CCC) terms.

**Methods:**

CNTRAM is a 2-stage retrieval-augmented framework that integrates dense embedding retrieval, retrieval-enhanced prompting, and few-shot LLM guidance to map free-text nursing records to standardized CCC terminology. Free-text records and their segments were embedded as subqueries to retrieve the most relevant CCC reference entries and annotated examples, which were merged to construct context windows. Each subquery was combined with its retrieved context using a predefined RAG prompt template that enforces CCC coding rules and a structured JSON schema and was then processed by an LLM to generate CCC outputs. A gold standard dataset of 100 intensive care unit nursing records was annotated by 3 senior nurses and finalized via consensus, with interrater reliability quantified using the Fleiss κ. Model performance was compared with traditional baselines (term frequency–inverse document frequency, Bidirectional Encoder Representations from Transformer, and fine-tuned Bidirectional Encoder Representations from Transformers model) and 4 LLMs (Mistral-7B, Qwen3-14B, Llama3.3-70B, and DeepSeek-R1) across no-RAG, zero-shot, and few-shot settings, using precision, recall, *F*_1_-score, and intersection over union (IoU) as metrics.

**Results:**

Interrater agreement was substantial, with Fleiss κ=0.6449 for diagnoses and κ=0.6180 for interventions. CNTRAM achieved substantial performance gains over all baseline approaches. For nursing diagnoses, DeepSeek-R1 with RAG+few-shot prompting achieved the best performance, with a precision of 0.7909, a recall of 0.7901, an *F*_1_-score of 0.7836, and an IoU of 0.7614. These results were significantly higher than those of traditional baselines (*F*_1_-score 0.0268‐0.2027), no-RAG LLMs (*F*_1_-score 0.0299‐0.0588), and RAG+zero-shot LLMs (*F*_1_-score 0.0716‐0.2160). For nursing interventions, the same configuration achieved a precision of 0.8453, a recall of 0.8504, an *F*_1_-score of 0.8413, and an IoU of 0.8097, outperforming traditional baselines (*F*_1_-score 0.1200‐0.2323), no-RAG LLMs (*F*_1_-score 0.0077‐0.0189), and RAG+zero-shot LLMs (*F*_1_-score 0.2744‐0.4461).

**Conclusions:**

This study developed CNTRAM, an LLM-based 2-stage RAG framework that combines dense embedding retrieval and few-shot prompting for CCC terminology mapping. Using DeepSeek-R1, CNTRAM outperformed baseline models, improved mapping accuracy, and provided a feasible solution for standardizing unstructured nursing data.

## Introduction

The widespread adoption of electronic health records (EHRs) has significantly enhanced the collection and use of clinical data [1], providing a solid foundation for data-driven research and continuous improvement of health care practices [2]. Within EHR systems, nursing records constitute a core data source, documenting nurses’ clinical observations, patient assessments, symptom responses, and nursing interventions delivered during routine care [3]. These records support clinical decision-making and care planning and also constitute an important primary data source for nursing research [4]. Despite the transition of nursing documentation from paper-based to electronic systems, most nursing records are still recorded in unstructured free-text form. This lack of structure substantially constrains their secondary use, semantic interoperability, and integration into clinical decision support systems [5].

To address these challenges, standardized nursing terminologies (SNTs) have been developed to enhance the consistency, comparability, and interoperability of nursing records [6,7]. Among them, the Clinical Care Classification (CCC) system is a comprehensive, coded nursing terminology designed specifically for the digital documentation of nursing care [8]. The CCC system comprises 176 nursing diagnoses and 201 core nursing interventions, organized into 21 care components covering physiological, functional, psychological, and health behavioral domains. By integrating nursing diagnoses, interventions, and outcomes within a unified structure, CCC provides a systematic framework for representing nursing practice [9]. Compared to the more complex and granular North American Nursing Diagnosis Association International (NANDA-I), Nursing Interventions Classification (NIC), or International Classification for Nursing Practice systems, the CCC system has a more compact semantic space, reducing conceptual ambiguity during the automatic mapping process [10]. A Chinese translation of the CCC system was released in 2018 to support its application in Chinese-language clinical settings, enabling standardized nursing documentation in Chinese hospitals and improving the interoperability of nursing data [11].

Mapping free-text nursing records to CCC terms enables the structured representation of nursing care processes, supports care quality assessment, and facilitates large-scale nursing research [12]. However, CCC-based annotation still relies heavily on manual coding, which is labor intensive, time consuming, and subject to interannotator variability, highlighting the urgent need for automated solutions that can reliably link unstructured nursing text to standardized terminology [13,14].

Recent advances in natural language processing and machine learning have substantially improved the automated structuring of unstructured clinical text [15-17]. Early rule-based and dictionary matching approaches were limited by heavy manual maintenance and poor generalizability to complex nursing language [18,19]. The advent of pretrained contextual models, such as Bidirectional Encoder Representations from Transformers (BERT) [20], enabled deeper semantic understanding and improved performance in tasks such as entity recognition, relation extraction, and text classification. Recently, large language models (LLMs) have further revolutionized the field, exhibiting strong capabilities in long-text comprehension, zero- and few-shot learning, and structured text generation [21]. Techniques such as retrieval-augmented generation (RAG) further enhance factual accuracy and interpretability by integrating external knowledge retrieval [22]. For example, a nursing-focused study showed that integrating a curated breast cancer nursing knowledge base with GPT-4 (OpenAI) improved the accuracy and clinical relevance of nursing responses while preserving empathetic communication [23].

Despite these technological advances, applications within nursing informatics remain limited. Most existing methods are rule driven, limited in scalability, or focused on general clinical text processing, while studies specifically targeting the automated identification of nursing diagnoses and interventions remain scarce. To address this gap, this study proposes an LLM-based framework for the automated extraction of nursing diagnoses and interventions aligned with the CCC system, aiming to reduce reliance on manual coding by nurses, enhance interoperability, and support data-driven decision-making in nursing practice.

## Methods

### Ethical Considerations

This study was approved by the ethics committee of the Chinese People’s Liberation Army General Hospital (S2024-767-01). Given the retrospective nature of this study, the requirement for written informed consent was waived by the ethics committee. No compensation was provided to participants. All data were fully anonymized before being included in the study. The study adhered to relevant ethical guidelines and protected the privacy and confidentiality of all patient records throughout the research process.

### Dataset

This study included 197,337 nursing records from the intensive care unit (ICU) of Chinese People’s Liberation Army General Hospital, covering the period from January 2023 to May 2025. These records consisted of free-text descriptions of time-stamped nursing assessments, interventions, and patient responses. All records were deidentified using a validated deidentification pipeline.

### Gold Standard Data Annotation

**Figure 1.**
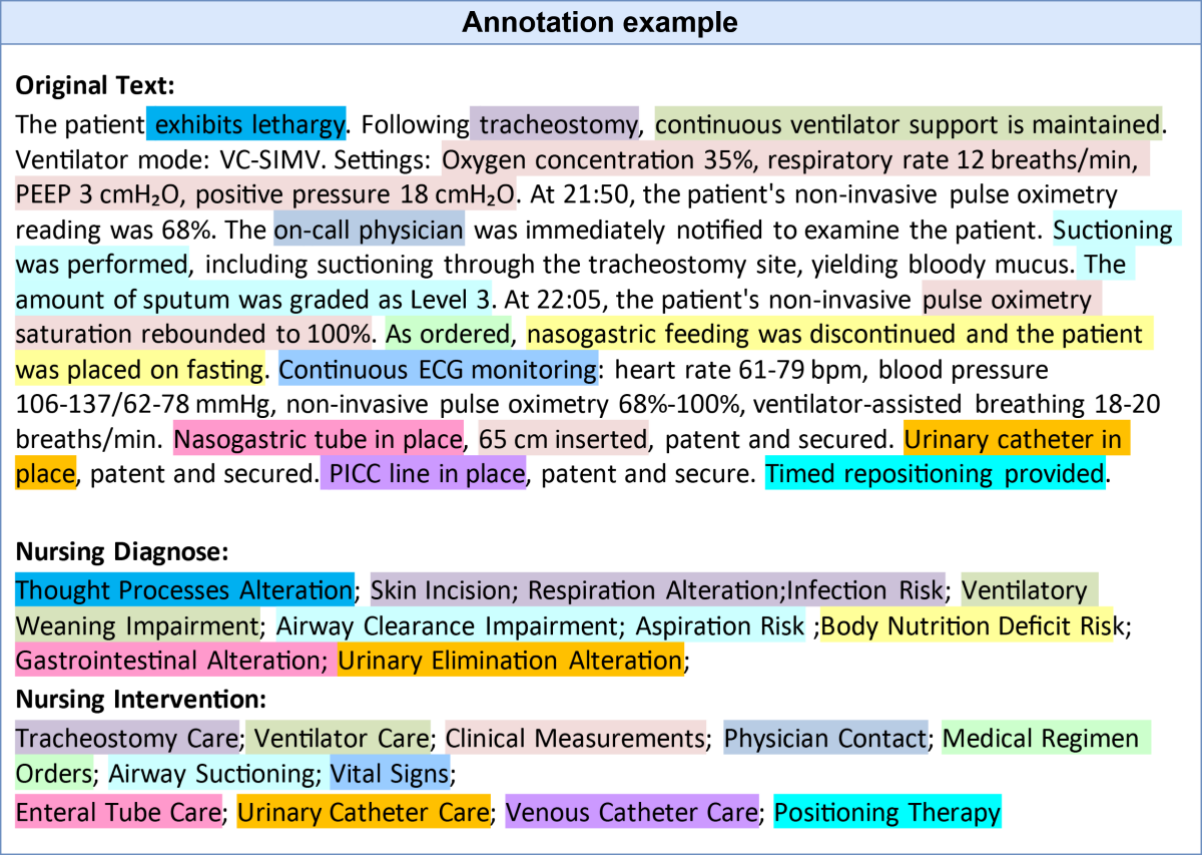
Annotation example. This figure shows how a segment of unstructured intensive care unit nursing text from a local hospital was manually annotated using Clinical Care Classification nursing diagnoses and interventions. Text segments with the same background color indicate the correspondence between the narrative content and the assigned nursing diagnosis or intervention.

To establish a gold standard dataset for model evaluation, 100 ICU nursing records were randomly selected for expert annotation. Three senior registered nurses, each with >5 years of ICU experience and prior familiarity with SNTs, independently annotated the nursing diagnoses and interventions for all samples. Before annotation, the annotators received a structured briefing on the CCC terminology, including term definitions, hierarchical structure, and mapping principles. Annotation was conducted in accordance with the official CCC terminology definitions and classification guidelines. Interrater reliability among the 3 annotators was assessed using the Fleiss κ, which indicated substantial agreement. The gold standard dataset was established through discussions among the 3 nurses to reach consensus on differing annotations. A representative annotated example is shown in Figure 1.

### Annotated Corpus

In total, 1500 nursing text samples were randomly selected from the full dataset to construct a manually annotated corpus. Two nursing postgraduate students, each with at least 3 months of ICU training, independently performed annotation after receiving standardized training based on the CCC terminology guidelines. The task involved identifying nursing diagnoses and interventions mentioned in each text and mapping them to the most appropriate CCC terms. When exact matches were unavailable, corresponding NANDA-I and NIC terms were applied as supplementary standards (Multimedia Appendix 1). In cases of disagreement, a senior ICU nurse adjudicated the final labels. To ensure data separation, the 100 expert-annotated evaluation records and the 1500 manually annotated corpus samples were sourced from distinct patients.

### CCC Nursing Terminology With Retrieval-Augmented Mapping

**Figure 2.**
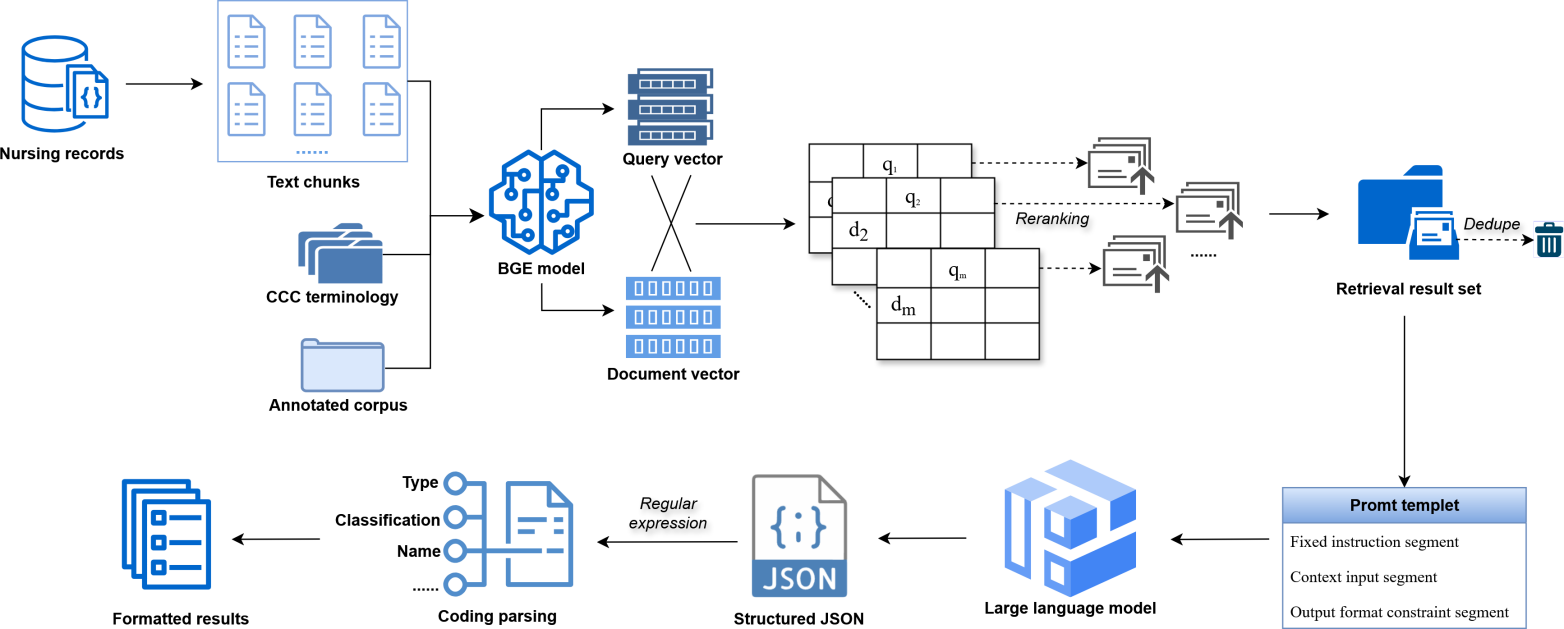
Clinical Care Classification (CCC) nursing terminology with retrieval-augmented mapping (CNTRAM) framework. This figure illustrates the 2-stage CNTRAM process for mapping free-text nursing records to standardized CCC diagnoses and interventions. Free-text records are segmented into text chunks, while the original records are retained as subqueries. All subqueries, together with CCC reference entries and annotated corpora, are embedded into 512-dimensional vectors using the BAAI (Beijing Academy of Artificial Intelligence) General Embedding–small-zh model. For each subquery, the top 5 most relevant entries are separately retrieved from CCC references and annotated corpora, then merged and deduplicated to construct context windows. Each subquery is combined with its context window to generate prompts under a predefined retrieval-augmented generation template that adheres to CCC coding guidelines and structured JSON output requirements. Prompts are fed into a locally deployed large language model, and the generated JSON outputs are parsed, cross-referenced against the CCC database for metadata validation, and formatted into standardized nursing coding results.

We developed CCC nursing terminology with retrieval-augmented mapping (CNTRAM), a 2-stage RAG framework, to map free-text nursing records to standardized CCC diagnoses and interventions (Figure 2). CNTRAM integrates dense embedding retrieval, retrieval-enhanced prompting, and few-shot LLM guidance to improve mapping accuracy and coverage.

In the first stage, free-text records were segmented into text chunks using Chinese and English punctuation, while the original records were retained as subqueries. Given the small scale of the CCC terminology set and its abundant clinically equivalent paraphrases, we prioritized a fine-grained semantic matching strategy. Specifically, we used the BAAI (Beijing Academy of Artificial Intelligence) General Embedding–small-zh model to embed each subquery, all CCC reference entries, and the full annotated corpus into 512-dimensional dense vectors. Semantic similarity between subqueries, reference entries, and annotated corpora was computed using matrix-based cosine scoring. In detail, the embedding vectors of subqueries and CCC reference entries were L2 normalized to unit norm. Subsequently, matrix multiplication was performed to compute the dot product of these normalized vectors, generating a similarity matrix of dimensions (number of subqueries×number of reference entries), where each element quantifies the semantic similarity between an individual subquery and a reference entry. Retrieval results from all subqueries were aggregated, and the unique entries remaining after duplicate removal were sorted by their similarity scores. To determine the retrieval window, we evaluated retrieval coverage via Recall@k (k=1, 3, 5, and 10). Recall@5 provided the optimal balance between retrieval sensitivity and computational cost and was adopted as the default setting (Multimedia Appendix 2). For each subquery, the top 5 most relevant entries were retrieved separately from both the CCC reference entries and the annotated corpus, merged, and deduplicated to construct context windows.

**Figure 3.**
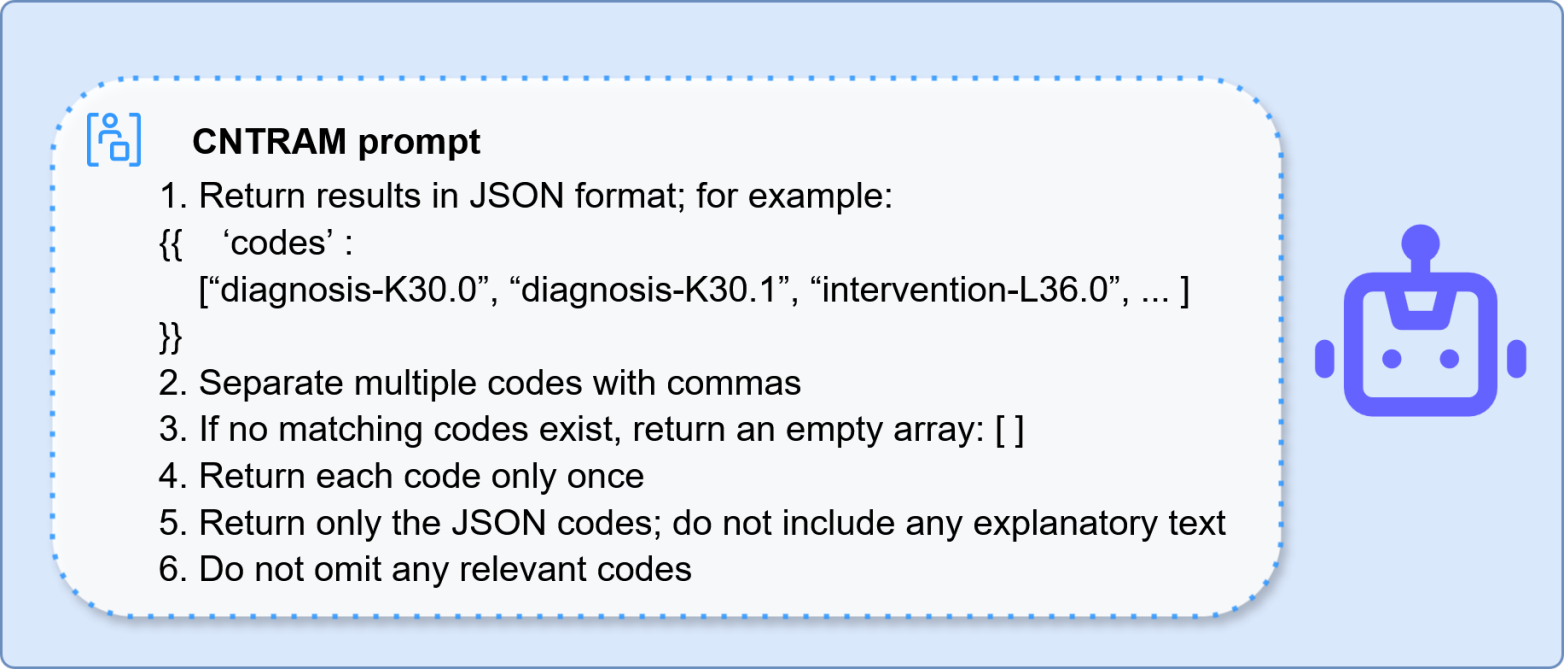
Clinical Care Classification (CCC) nursing terminology with retrieval-augmented mapping (CNTRAM) prompt. This figure shows the standardized prompt format used in CNTRAM and the expected JSON-based output. The prompt specifies that the model should return CCC nursing diagnosis and intervention codes as a JSON array, without explanatory text or duplication; an empty array is returned if no valid codes are identified.

In the second stage, prompts were generated by combining each subquery with its retrieved context following a predefined RAG template. This template enforces CCC coding guidelines, which incorporate the supplementary NANDA-I and NIC terms, and defines a structured JSON output schema (Figure 3). These prompts were then processed by a locally deployed LLM, which generated structured JSON outputs [24]. The resulting JSON was parsed with regular expressions, cross-referenced against the term database for metadata validation, and formatted into a standardized structure suitable for downstream analysis.

### Experiments

To establish comparative baselines and evaluate CNTRAM’s relative performance, multiple models were implemented, encompassing traditional statistical methods, pretrained language models, and LLMs.

Free-text inputs were transformed into term frequency–inverse document frequency (TF-IDF) vectors [25], and candidate CCC terms were ranked by cosine similarity. This served as a lexical-matching baseline reflecting rule-driven coding.

Contextual embeddings were generated using BERT [20]. Semantic similarity between text segments and CCC entries was calculated via cosine distance. This represented a rule-driven baseline with contextual semantic awareness. Additionally, BERT was fine-tuned on the annotated corpus of 1500 records to provide a supervised baseline for comparison.

Llama3.3-70B (Meta Platforms Inc) [26], Qwen3-14B (Alibaba Group) [27], Mistral-7B (Mistral AI) [28], and DeepSeek-R1 (DeepSeek) [29] were evaluated under zero-shot and few-shot prompting to test their intrinsic ability to align free-text nursing notes with CCC terms based solely on coding rules. Additionally, no-RAG experiments were conducted for each LLM to serve as a comparison.

### Prompting Strategies

To examine the influence of contextual retrieval and example-based guidance, 2 prompting strategies were compared under the CNTRAM framework.

First, zero-shot prompting was used; for each generated subquery derived from both the segmented text and the original records, the top 5 most semantically relevant CCC coding rules were retrieved and incorporated into the prompt [30]. No example demonstrations were included.

For few-shot prompting, few-shot examples were selected by retrieving the top 5 most semantically relevant examples for each subquery generated from both the segmented text and the original records [31]. These examples were combined with retrieved CCC coding rules, which were generated following the same approach as zero-shot prompting, to form the final prompt. We further assessed the contribution of few-shot examples without retrieval using 20 static examples in the prompt.

### Evaluation Metrics

Model performance was evaluated using precision, recall, *F*_1_-score, and intersection over union (IoU), with each nursing record treated as a set of terms. CCC terms and supplementary NANDA-I and NIC terms were included in the evaluation. As a single record may contain multiple clinically related diagnoses and interventions, IoU was additionally used to more comprehensively assess the model’s ability to identify all nursing terms, capturing their combinatorial characteristics. IoU is defined as the ratio of the intersection to the union of the predicted and gold standard term sets.

For a given record *i*, let *G*_*i*_ denote the gold standard term set and *P*_*i*_ denote the predicted term set. In this notation, “|⋅|” denotes set cardinality, “\” denotes set difference, and “∩” denotes intersection. The evaluation components were defined as follows:

$TP_{i}/G_{i}\cap P_{i}/\;,FP_{i}=/P_{i}\setminus G_{i}/\;,FN_{i}=/G_{i}\setminus P_{i}/$

Metrics for each sample were calculated as follows:


$$Precision_{i}=\frac{TP_{i}}{TP_{i}+FP_{i}},Recall_{i}=\frac{TP_{i}}{TP_{i}+FN_{i}},F1_{i}=\frac{2\cdot Precision_{i}\cdot Recall_{i}}{Precision_{i}+Recall_{i}}$$

and

$IoU_{i}=\frac{TP_{i}}{TP_{i}+FP_{i}+FN_{i}}$

where *TP*_*i*_ is the number of correctly predicted terms in record *i*, *FP*_*i*_ is the number of predicted terms not in the gold standard set, and *FN*_*i*_ is the number of gold standard terms missed by the prediction. Overall performance was calculated as the macroaverage of each metric across all records.

## Results

### Characteristics of the Dataset

This dataset contained 197,337 records from 880 patients, representing an older cohort (median age 67, IQR 57-74 years) with a wide range of ICU lengths of stay (median 1 day, IQR 0-6 days), as shown in Table 1.

**Table 1.** Dataset characteristics (N=880).

Characteristics	Values
Nursing note entries, n	197,337
Age (years), median (IQR)	67 (57-74)
Female sex, n (%)	287 (32.6)
Length of stay in the intensive care unit (days), median (IQR)	1 (0‐6)

### Annotator Agreement and Coverage of CCC Terminology

Interrater reliability was evaluated using the Fleiss κ, which measures the degree of agreement among multiple annotators beyond chance. The results showed substantial consistency for both categories, with κ=0.65 for nursing diagnoses and κ=0.62 for nursing interventions (Table 2). These results indicate that the 3 nurses demonstrated a consistent application of CCC terminology during independent annotation, providing a solid foundation for constructing the final consensus-based reference standard used for model evaluation. In terms of terminology coverage, a total of 65 unique nursing diagnoses were observed in the annotated 100-record test set, corresponding to 36.9% of the 176 CCC diagnosis codes. For nursing interventions, 106 unique codes were present, representing 52.7% of the 201 CCC intervention codes.

**Table 2.** Fleiss κ for interrater agreement among 3 senior registered nurses.

Items	Observed agreement	Expected agreement	1 minus expected agreement	Fleiss κ
Nursing diagnoses	0.9886	0.9679	0.0321	0.6449
Nursing interventions	0.9830	0.9555	0.0445	0.6180

### Model Performance

The CNTRAM framework demonstrated comprehensive superiority over TF-IDF, BERT, fine-tuned BERT, and zero-shot LLMs in mapping nursing records to standardized CCC terminology by integrating retrieval-enhanced and few-shot prompting. For nursing diagnoses (Table 3), the best-performing model was DeepSeek-R1. It achieved a precision of 0.7909, a recall of 0.7901, an *F*_1_-score of 0.7836, and an IoU of 0.7614. All other approaches showed lower results across all metrics. Among traditional methods and language models, the *F*_1_-score ranged from 0.0268 (BERT) to 0.5253 (Qwen3-14B with RAG+few-shot), and the IoU values ranged from 0.0142 (BERT) to 0.4987 (Qwen3-14B with RAG+few-shot).

**Table 3.** Model performance for mapping nursing diagnoses.

Models and prompting strategies	Precision	Recall	*F*_1_-score	Intersection over union
Term frequency–inverse document frequency	0.0738	0.2733	0.0856	0.0685
BERT^a^	0.0155	0.2460	0.0268	0.0142
Fine-tuned BERT	0.2223	0.2233	0.2027	0.1798
No RAG^b^				
Mistral-7B	0.0396	0.0316	0.0299	0.0252
Qwen3-14B	0.0482	0.0406	0.0412	0.0394
Llama3.3-70B	0.0570	0.0511	0.0515	0.0511
DeepSeek-R1	0.0601	0.0582	0.0588	0.0580
RAG+zero-shot				
Mistral-7B	0.0727	0.0706	0.0716	0.0704
Qwen3-14B	0.0992	0.0804	0.0849	0.0800
Llama3.3-70B	0.1044	0.0906	0.0937	0.0903
DeepSeek-R1	0.2310	0.2241	0.2160	0.2003
RAG+few-shot				
Mistral-7B	0.4657	0.4374	0.4298	0.3914
Qwen3-14B	0.5614	0.5213	0.5253	0.4987
Llama3.3-70B	0.4819	0.4351	0.4455	0.4245
DeepSeek-R1	*0.7909*	*0.7901*	*0.7836*	*0.7614*

a BERT: Bidirectional Encoder Representations from Transformers.

b RAG: retrieval-augmented generation.

For nursing interventions (Table 4), DeepSeek-R1 again showed the best overall performance, with a precision of 0.8453, a recall of 0.8504, an *F*_1_-score of 0.8413, and an IoU of 0.8097. Other traditional methods and language model setups exhibited lower performance, with *F*_1_-score varying from 0.0077 (Mistral-7B with no RAG) to 0.5557 (Qwen3-14B with RAG+few-shot) and IoU values ranging from 0.0071 (Mistral-7B with no RAG) to 0.5072 (Qwen3-14B with RAG+few-shot).

**Table 4.** Model performance for mapping nursing interventions.

Model and prompting strategies	Precision	Recall	*F*_1_-score	Intersection over union
Term frequency–inverse document frequency	0.2112	0.5508	0.2323	0.1597
BERT^a^	0.0818	0.3854	0.1200	0.0681
Fine-tuned BERT	0.2297	0.1686	0.1723	0.1290
No RAG^b^				
Mistral-7B	0.0079	0.0506	0.0077	0.0071
Qwen3-14B	0.0129	0.0148	0.0132	0.0129
Llama3.3-70B	0.0142	0.0131	0.0134	0.0130
DeepSeek-R1	0.0203	0.0182	0.0189	0.0166
RAG+zero-shot				
Mistral-7B	0.3885	0.2411	0.2744	0.1907
Qwen3-14B	0.4874	0.4147	0.4298	0.3174
Llama3.3-70B	0.4074	0.3932	0.3849	0.2782
DeepSeek-R1	0.4973	0.4903	0.4461	0.3563
RAG+few-shot				
Mistral-7B	0.5218	0.4049	0.4299	0.3561
Qwen3-14B	0.6018	0.5458	0.5557	0.5072
Llama3.3-70B	0.5757	0.4844	0.5052	0.4368
DeepSeek-R1	*0.8453*	*0.8504*	*0.8413*	*0.8097*

a BERT: Bidirectional Encoder Representations from Transformers.

b RAG: retrieval-augmented generation.

### Prompting Strategies

**Figure 4.**
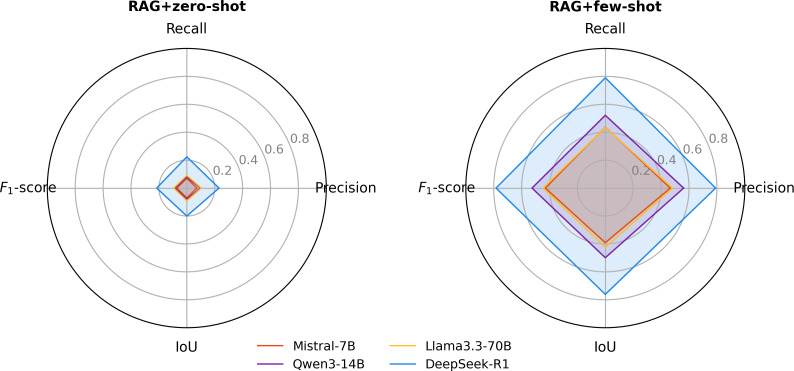
Model performance for nursing diagnosis recognition under different prompting strategies: (left) retrieval-augmented generation (RAG)+zero-shot prompting and (right) RAG+few-shot prompting. This figure shows the performance of different large language models (Mistral-7B, Qwen3-14B, Llama3.3-70B, and DeepSeek-R1) under zero-shot and few-shot prompting conditions in recognizing Clinical Care Classification nursing diagnoses from free-text intensive care unit nursing records. Metrics include precision, recall, *F*_1_-score, and intersection over union (IoU).

**Figure 5.**
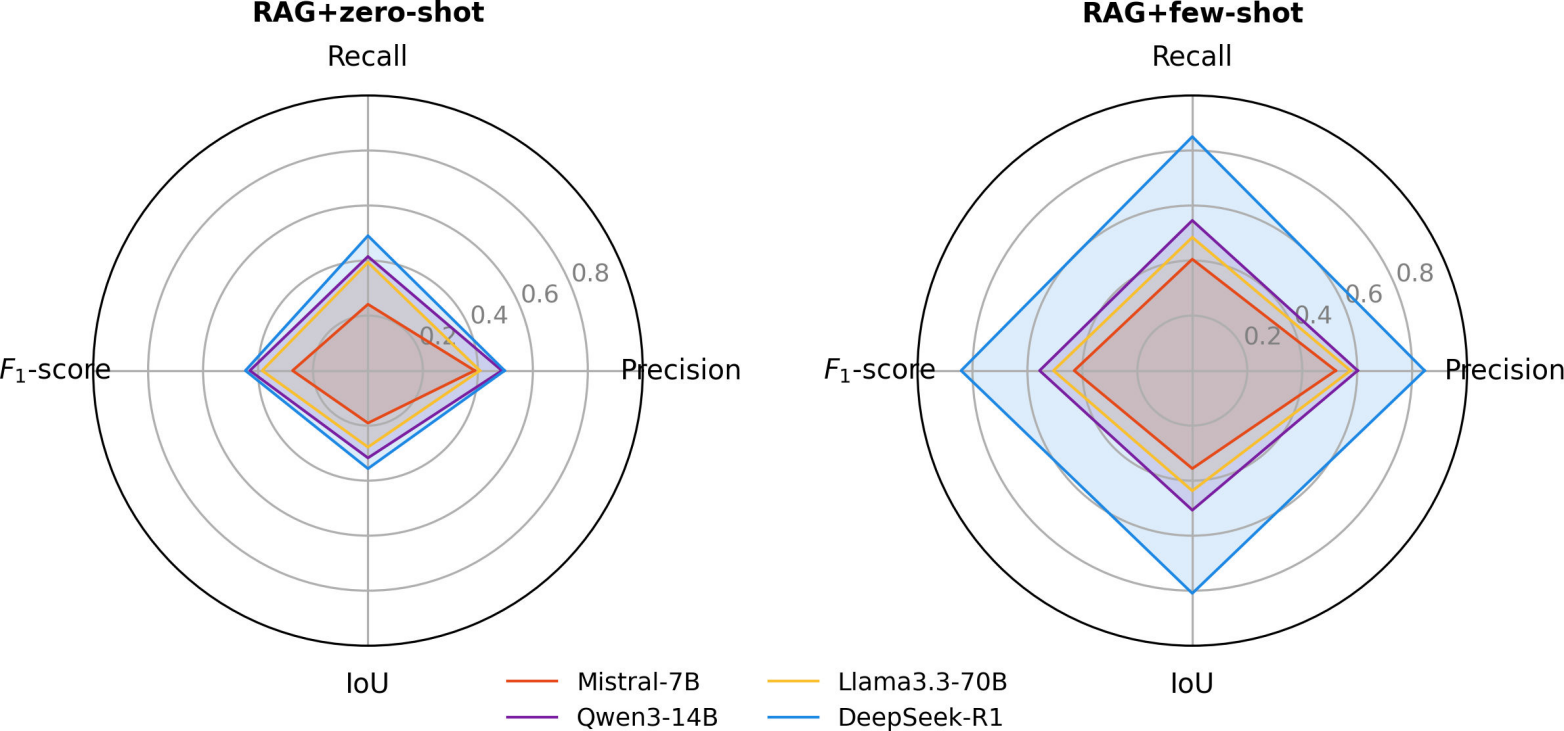
Model performance for nursing intervention recognition under different prompting strategies: (left) retrieval-augmented generation (RAG)+zero-shot prompting and (right) RAG+few-shot prompting. This figure shows the performance of different large language models (Mistral-7B, Qwen3-14B, Llama3.3-70B, and DeepSeek-R1) under zero-shot and few-shot prompting conditions in recognizing Clinical Care Classification nursing diagnoses from free-text intensive care unit nursing records. Metrics include precision, recall, *F*_1_-score, and intersection over union (IoU).

We evaluated the impact of prompting strategies by comparing zero-shot and few-shot setups across all models. Few-shot prompting consistently improved performance for both nursing diagnoses and interventions. For nursing diagnoses (Figure 4), *F*_1_-score across the 4 models ranged from 0.0716 to 0.216 under RAG+zero-shot and increased to a range of 0.4298 to 0.7836 under RAG+few-shot. Corresponding IoU values ranged from 0.0704 to 0.2003 with RAG+zero-shot and from 0.3914 to 0.7614 with RAG+few-shot. For nursing interventions (Figure 5), *F*_1_-score ranged from 0.2744 to 0.4461 with RAG+zero-shot and improved to 0.4299‐0.8413 with RAG+few-shot. IoU values ranged from 0.1907 to 0.3563 with RAG+zero-shot and from 0.3561 to 0.8097 with RAG+few-shot. In both tasks, DeepSeek-R1 with RAG+few-shot achieved the best performance. Ablation results for the no-RAG+few-shot configuration are provided in Multimedia Appendix 3.

## Discussion

### Principal Findings

In this study, we developed CNTRAM, a retrieval-augmented terminology mapping framework integrating dense embedding retrieval, retrieval-enhanced prompting, and few-shot LLM guidance to automatically identify nursing diagnoses and interventions from free-text nursing records based on standardized CCC terminology. Our findings demonstrate that combining RAG with carefully designed few-shot examples markedly improves mapping accuracy compared with TF-IDF, BERT, fine-tuned BERT, and zero-shot LLMs. Specifically, our CNTRAM framework, based on DeepSeek-R1, achieved substantial performance improvements in both nursing diagnosis and intervention mapping. These results align with growing evidence that retrieval grounding improves the factual consistency of clinical text generation models [32-34].

The core challenge addressed by CNTRAM stems from the semantic characteristics inherent in nursing terminology. Nursing diagnoses are typically abstract conceptual constructs requiring inference, demanding that nurses synthesize multiple clues and arrive at diagnoses through reasoning [35,36]. Many diagnoses represent higher-order concepts that do not explicitly appear in nursing narratives. In contrast, nursing interventions are more concrete, operationalized, and linguistically salient, typically expressed through explicit action verbs such as “monitor,” “turn,” or “assess.” Previous analyses of CCC semantic structure have also noted that intervention concepts are more operationalized and lexically consistent, while diagnostic concepts may overlap semantically and contain broader conceptual boundaries, complicating automated classification [37,38]. These semantic differences likely explain the higher precision and *F*_1_-scores observed in mapping nursing interventions and diagnoses in this study. This also underscores the need for technical approaches that support abstraction and reasoning capabilities during nursing terminology standardization.

On the basis of the professional attributes of nursing terminology, the performance advantages of the CNTRAM constructed in this study are primarily demonstrated through its 3 components. First, the RAG module retrieves contextually relevant CCC entries for each subquery, providing semantic grounding that reduces ambiguity and stabilizes concept alignment [39]. This design is consistent with growing evidence that retrieval augmentation improves reliability in medical question answering and EHR summarization [40]. Second, the 2-stage generation structure enforces strict adherence to CCC coding rules while producing stable JSON outputs that support downstream analysis. Finally, few-shot examples offer essential syntactic and semantic anchors for interpreting abstract or compositionally complex nursing expressions such as “airway clearance impairment,” “aspiration risk,” “infection risk,” “venous catheter care,” and “positioning therapy.” Evidence from biomedical natural language processing similarly shows that combining retrieval with example-guided prompting markedly improves terminology normalization when annotated data are scarce [41].

Beyond task-level performance, CNTRAM maintains methodological continuity with broader concept normalization research while advancing terminology standardization in nursing. Within medical concept normalization, hybrid approaches that integrate rule-based matching, multilevel semantic similarity, machine learning, or multistage LLM workflows have become increasingly prominent for Unified Medical Language System or Systematized Nomenclature of Medicine–Clinical Terms normalization [42-44]. Meanwhile, nursing informatics literature has long emphasized that SNTs enhance interoperability and enable meaningful analysis of nursing patterns [45]. Previous cross-mapping studies demonstrated the feasibility of aligning narrative nursing documentation with vocabularies such as NANDA-I and NIC, although manual mapping workflows proved time consuming and inconsistent across institutions [46,47]. By demonstrating that retrieval-enhanced LLMs can adapt effectively to nursing-specific terminology systems and documentation practices, CNTRAM extends these research directions and offers a practical pathway for automated nursing terminology mapping in real-world environments. Additionally, in this study, the use of LLMs with varying parameter scales showed differing recognition performance. Among them, DeepSeek-R1 outperformed the other models, likely due to its stronger reasoning ability, instruction-following capacity, and better adaptation to Chinese clinical text. This reasoning advantage allows it to handle ambiguous or complex medical and nursing expressions more effectively [29]. Coupled with reliable instruction-following capacity, this ensures accurate mapping within the constrained output schema [48]. Moreover, its specialization in Chinese clinical data further enhances its ability to accurately map nursing terminology, which is essential given the unique linguistic and contextual features of Chinese clinical language [49].

From a nursing practice perspective, CNTRAM offers a scalable method for converting unstructured nursing records into structured, analyzable data. Identifying nursing practice features, such as nursing diagnoses and interventions, within practitioners’ observation records helps validate the value of SNT for enhancing interoperability and the applicability of nursing data [45,50,51]. By identifying patients’ nursing problems and clarifying corresponding nursing actions, CNTRAM can provide a clinically interpretable basis for risk stratification and outcome assessment. Recent observational evidence further supports this direction. Studies from Italian pediatric [52,53] and adult oncology settings [54] have shown that CCC-based nursing data can capture nursing care complexity and are associated with intrahospital and ICU transfers, diagnosis related group–based medical complexity, and length of stay. These findings reinforce the potential of structured nursing data to reflect patient risk and care demand. Accordingly, the CCC-coded information produced by CNTRAM offers a structured foundation that can be incorporated into future analyses of complexity, outcomes, and resource use. In addition, the high IoU score achieved in this study indicates the model’s ability to effectively represent comprehensive sets of nursing diagnoses and interventions, reflecting the combinatorial nature of the nursing process. Notably, nursing diagnoses such as risk of infection and risk of aspiration require comprehensive judgment based on multiple clinical conditions and are prone to certain recognition errors [55]. Generalized nursing interventions, such as clinical measurements and physical examination, may have mapping biases due to unclear classification granularity [56]. Misclassification of nursing diagnoses can affect nurses’ risk stratification assessment of patients, while misclassification of nursing interventions may disturb the calculation of nursing workload and the correlation between intervention frequency and patient risk [57]. In practical application, CNTRAM outputs can serve as an aid for clinical decision support and documentation, but they still require judgment and review by clinical nurses.

### Limitations and Future Work

However, this study has several limitations. First, the analysis was based on data from a single ICU with a relatively limited dataset, and the test set covered only a subset of commonly used CCC codes. This partial coverage constrains our ability to generalize the findings to the full CCC terminology system. Second, although few-shot prompting improved model performance, it introduced a dependence on manually curated examples. Such reliance may lead to sampling bias. Third, as highlighted in recent health care RAG literature, robust evaluation frameworks are essential to mitigate issues related to bias, trust, and privacy [58]. Future research could further validate the generalization capability of CNTRAM through multicenter and multilingual data verification. Additionally, the flexibility of CNTRAM to adapt to different nursing terminology systems, such as NANDA-I, NIC, and International Classification for Nursing Practice, could be examined. This would involve integrating diverse domain knowledge resources into the RAG framework to enhance semantic granularity and expand the coverage of terminology mapping [59]. Furthermore, embedding CNTRAM into EHR systems for real-time semantic standardization could provide actionable support for nursing practice and clinical decision-making.

### Conclusions

In this study, we developed CNTRAM, an LLM-based 2-stage RAG framework designed to map free-text nursing records to the CCC terminology. CNTRAM combines dense embedding retrieval and few-shot prompting. Using DeepSeek-R1 as its backbone LLM, this framework outperformed traditional methods, no-RAG models, and zero-shot prompting approaches. The results demonstrate that the integration of RAG, few-shot learning, and LLMs improves mapping accuracy and offers a feasible solution for mapping unstructured nursing data to standardized terminologies.

## Supplementary material

10.2196/89850 Multimedia Appendix 1 Supplementary terms.

10.2196/89850 Multimedia Appendix 2 Recall@k Values for Different Retrieval Window k

10.2196/89850 Multimedia Appendix 3 Performance of large language models under “No retrieval-augmented generation” and “Few-shot prompting” for nursing diagnosis and intervention tasks.

## Abbreviations

BAAI: Beijing Academy of Artificial Intelligence
BERT: Bidirectional Encoder Representations from Transformers
CCC: Clinical Care Classification
CNTRAM: Clinical Care Classification nursing terminology with retrieval-augmented mapping
EHR: electronic health record
ICU: intensive care unit
IoU: intersection over union
LLM: large language model
NANDA-I: North American Nursing Diagnosis Association International
NIC: Nursing Interventions Classification
RAG: retrieval-augmented generation
SNT: standardized nursing terminology
TF-IDF: term frequency–inverse document frequency

## References

[1] Opmeer BC (2016). Electronic health records as sources of research data. JAMA.

[2] Anderson C, Kaul M, Gullapalli N, Pitani S (2023). Electronic health records and clinical documentation in medical residency programs: preparing residents to become master clinicians. J Am Med Inform Assoc.

[3] Salanterä S (2015). Advanced use of electronic health records: the depth of nursing notes. Nurs Res.

[4] von Gerich H, Moen H, Peltonen LM (2022). Identifying nursing sensitive indicators from electronic health records in acute cardiac care-towards intelligent automated assessment of care quality. J Nurs Manag.

[5] Macieira TGR, Chianca TCM, Smith MB (2019). Secondary use of standardized nursing care data for advancing nursing science and practice: a systematic review. J Am Med Inform Assoc.

[6] Monsen KA, Heermann L, Dunn-Lopez K (2023). FHIR-up! Advancing knowledge from clinical data through application of standardized nursing terminologies within HL7® FHIR®. J Am Med Inform Assoc.

[7] Zhang T, Wu X, Peng G (2021). Effectiveness of standardized nursing terminologies for nursing practice and healthcare outcomes: a systematic review. Int J Nurs Knowl.

[8] Saba VK, Taylor SL (2007). Moving past theory: use of a standardized, coded nursing terminology to enhance nursing visibility. Comput Inform Nurs.

[9] Feng RC, Tseng KJ, Yan HF, Huang HY, Chang P (2013). A preliminary study on the use of clinical care classification in nursing documentation data sets. Comput Methods Programs Biomed.

[10] Kang MJ, Dykes PC, Korach TZ (2020). Identifying nurses’ concern concepts about patient deterioration using a standard nursing terminology. Int J Med Inform.

[11] Clinical nursing terminology index. Zhongwei Nursing Information Research Institute.

[12] Feng RC, Tseng KJ, Yan HF, Huang HY, Chang P (2012). Capability of using clinical care classification system to represent nursing practice in acute setting in Taiwan. NI 2012 (2012).

[13] Choi M, Kim Y, Kim Y (2024). Development of a mapping table for nursing notes based on nurses’ concerns in ICU patients. Stud Health Technol Inform.

[14] Torres FBG, Gomes DC, Hino AAF, Moro C, Cubas MR (2020). Comparison of the results of manual and automated processes of cross-mapping between nursing terms: quantitative study. JMIR Nurs.

[15] Elvas LB, Almeida A, Ferreira JC (2025). Natural language processing in medical text processing: a scoping literature review. Int J Med Inform.

[16] Hossain E, Rana R, Higgins N (2023). Natural language processing in electronic health records in relation to healthcare decision-making: a systematic review. Comput Biol Med.

[17] Korach ZT, Cato KD, Collins SA (2019). Unsupervised machine learning of topics documented by nurses about hospitalized patients prior to a rapid-response event. Appl Clin Inform.

[18] Redjdal A, Novikava N, Kempf E, Bouaud J, Seroussi B (2024). Leveraging rule-based NLP to translate textual reports as structured inputs automatically processed by a clinical decision support system. Stud Health Technol Inform.

[19] Lou Y, Zhu X, Tan K (2023). Dictionary-based matching graph network for biomedical named entity recognition. Sci Rep.

[20] Nishigaki D, Suzuki Y, Wataya T (2023). BERT-based transfer learning in sentence-level anatomic classification of free-text radiology reports. Radiol Artif Intell.

[21] Xiao H, Zhou F, Liu X (2025). A comprehensive survey of large language models and multimodal large language models in medicine. Inf Fusion.

[22] Ke YH, Jin L, Elangovan K (2025). Retrieval augmented generation for 10 large language models and its generalizability in assessing medical fitness. NPJ Digit Med.

[23] Xu R, Hong Y, Zhang F, Xu H (2024). Evaluation of the integration of retrieval-augmented generation in large language model for breast cancer nursing care responses. Sci Rep.

[24] JSON Output. DeepSeek API Docs.

[25] Bafna P, Pramod D, Vaidya A Document clustering: TF-IDF approach.

[26] Grattafiori A, Dubey A, Jauhri A, Pandey A, Kadian A, Al-Dahle A (2024). The Llama 3 herd of models. arXiv.

[27] Yang A, Li A, Yang B, Zhang B, Hui B, Zheng B (2025). Qwen3 technical report. arXiv.

[28] Jiang AQ, Sablayrolles A, Mensch A, Bamford C, Chaplot DS (2023). Mistral 7B. arXiv.

[29] Guo D, Yang D, Zhang H (2025). DeepSeek-R1 incentivizes reasoning in LLMs through reinforcement learning. Nature.

[30] Levy O, Seo M, Choi E, Zettlemoyer L Zero-shot relation extraction via reading comprehension.

[31] Parnami A, Lee M (2022). Learning from few examples: a summary of approaches to few-shot learning. ArXiv.

[32] Li M, Zhan Z, Yang H (2025). Benchmarking retrieval-augmented large language models in biomedical NLP: application, robustness, and self-awareness. Sci Adv.

[33] Koga S, Ono D, Obstfeld A (2025). Retrieval-augmented generation versus document-grounded generation: a key distinction in large language models. J Pathol Clin Res.

[34] Alkhalaf M, Yu P, Yin M, Deng C (2024). Applying generative AI with retrieval augmented generation to summarize and extract key clinical information from electronic health records. J Biomed Inform.

[35] Pirret AM, Neville SJ, La Grow SJ (2015). Nurse practitioners versus doctors diagnostic reasoning in a complex case presentation to an acute tertiary hospital: a comparative study. Int J Nurs Stud.

[36] Smith SK, Benbenek MM, Bakker CJ, Bockwoldt D (2022). Scoping review: diagnostic reasoning as a component of clinical reasoning in the U.S. primary care nurse practitioner education. J Adv Nurs.

[37] Fine S, Chaudhri A, Englebright J, Dan Roberts W (2023). Nursing process, derived from the clinical care classification system components, as an earlier indicator of nursing care during a pandemic. Int J Med Inform.

[38] Jansen K, Kim TY, Coenen A, Saba V, Hardiker N (2016). Harmonising nursing terminologies using a conceptual framework. Stud Health Technol Inform.

[39] Zhong M, Wu Z, Honda N (2024). Deep learning based dense retrieval: a comparative study. ArXiv.

[40] Liu S, McCoy AB, Wright A (2025). Improving large language model applications in biomedicine with retrieval-augmented generation: a systematic review, meta-analysis, and clinical development guidelines. J Am Med Inform Assoc.

[41] Kresevic S, Giuffrè M, Ajcevic M, Accardo A, Crocè LS, Shung DL (2024). Optimization of hepatological clinical guidelines interpretation by large language models: a retrieval augmented generation-based framework. NPJ Digit Med.

[42] Chen L, Fu W, Gu Y (2020). Clinical concept normalization with a hybrid natural language processing system combining multilevel matching and machine learning ranking. J Am Med Inform Assoc.

[43] Yoon D, Han C, Kim DW (2024). Redefining health care data interoperability: empirical exploration of large language models in information exchange. J Med Internet Res.

[44] Afshar M, Gao Y, Gupta D, Croxford E, Demner-Fushman D (2024). On the role of the UMLS in supporting diagnosis generation proposed by large language models. J Biomed Inform.

[45] Dunn Lopez K, Heermann Langford L, Kennedy R (2023). Future advancement of health care through standardized nursing terminologies: reflections from a Friends of the National Library of Medicine workshop honoring Virginia K. Saba. J Am Med Inform Assoc.

[46] D’Agostino F, Zeffiro V, Vellone E (2020). Cross-mapping of nursing care terms recorded in Italian hospitals into the standardized NNN terminology. Int J Nurs Knowl.

[47] Juvé Udina ME, Gonzalez Samartino M, Matud Calvo C (2012). Mapping the diagnosis axis of an interface terminology to the NANDA international taxonomy. ISRN Nurs.

[48] Chen X, Liao B, Qi J, Eustratiadis P, Monz C, Bisazza A The SIFo benchmark: investigating the sequential instruction following ability of large language models.

[49] Yao Z, Duan L, Xu S, Chi L, Sheng D (2025). Performance of large language models in the non-English context: qualitative study of models trained on different languages in Chinese medical examinations. JMIR Med Inform.

[50] Jedwab RM, Holzhauser K, Raghunathan K (2024). Applicability and benefits of standardised nursing terminology in Australia: a scoping review. Collegian.

[51] Ranegger R, Baumberger D, Grgic P, Jagfeld G (2024). Semantic interoperability of nursing data - mapping an interface terminology to SNOMED CT. Stud Health Technol Inform.

[52] Cesare M, D’Agostino F, Sebastiani E, Damiani G, Cocchieri A, Nursing And Public Health Group (2025). Deciphering the link between diagnosis-related group weight and nursing care complexity in hospitalized children: an observational study. Children (Basel).

[53] Cesare M, Cocchieri A (2025). Can an increase in nursing care complexity raise the risk of intra-hospital and intensive care unit transfers in children? A retrospective observational study. J Pediatr Nurs.

[54] Cesare M, Magliozzi E, D’Agostino F, Zeffiro V, Cocchieri A (2025). Prevalence and accuracy of nursing diagnoses in patients with malignant bronchial and lung cancer: a retrospective observational study. Eur J Oncol Nurs.

[55] Hasegawa T, Ogasawara C, Katz EC (2007). Measuring diagnostic competency and the analysis of factors influencing competency using written case studies. Int J Nurs Terminol Classif.

[56] Coenen A, Ryan P, Sutton J (1997). Mapping nursing interventions from a hospital information system to the Nursing Interventions Classification (NIC). Nurs Diagn.

[57] Müller-Staub M, Lavin MA, Needham I, van Achterberg T (2006). Nursing diagnoses, interventions and outcomes - application and impact on nursing practice: systematic review. J Adv Nurs.

[58] Amugongo LM, Mascheroni P, Brooks S, Doering S, Seidel J (2025). Retrieval augmented generation for large language models in healthcare: a systematic review. PLOS Digit Health.

[59] Zhao X, Liu S, Yang SY, Miao C MedRAG: enhancing retrieval-augmented generation with knowledge graph-elicited reasoning for healthcare copilot.

[60] GitHub.

