# Supplementary material for: Automated Identification of Nursing Diagnoses and Interventions From Nursing Records Using a Retrieval-Augmented Large Language Model Approach: Quantitative Study
Source: J Med Internet Res. 2026 Apr 29;28:e89850. doi: 10.2196/89850 (PMC13128066; doi:10.2196/89850)
Supplement: Multimedia Appendix 1 [file jmir-v28-e89850-s001.docx]

Table S1 Supplementary Term

| Term Type | Classification | Classification code | Encoding | Term Name | Definition | Original code |
| --- | --- | --- | --- | --- | --- | --- |
| Nursing Diagnosis | Safety | N | 33.7 | Risk of Thrombosis | Blood clots may form and block blood vessels; these clots may rupture and lodge in another vessel, potentially causing harm to health | NANDA-I 00291 |
| Nursing Intervention | Respiratory | L | 37.1 | Airway Suctioning | Insert the suction catheter into the patient’s mouth and/or trachea to clear respiratory secretions | NIC  3160 |
| Nursing Intervention | Tissue Perfusion | S | 70.1 | Blood Products Administration | Administer blood or blood products to the patient and monitor their response | NIC  4030 |
| Nursing Intervention | Physical Regulation | K | 30.2 | Fever Treatment | Managing elevated body temperature caused by non-environmental factors | NIC  3740 |
| Nursing Intervention | Safety | N | 50.0 | Embolus Precautions | Reducing the risk of thrombosis in patients at risk of thrombosis | NIC  4110 |
| Nursing Intervention | Respiratory | L | 37.2 | Airway Insertion and Stabilization | Insert or assist in the insertion and securing of an artificial airway | NIC  3120 |
| Nursing Intervention | Metabolic | I | 27.1 | Hyperglycemia Management | Prevention and treatment of hyperglycaemia | NIC  2120 |
| Nursing Intervention | Cognitive/  Neuro | D | 78.1 | Sedation management | Administer sedatives, monitor the patient’s response, and provide necessary physiological support during diagnosis or treatment. | NIC  2260 |
