# Supplementary material for: Automated Identification of Nursing Diagnoses and Interventions From Nursing Records Using a Retrieval-Augmented Large Language Model Approach: Quantitative Study
Source: J Med Internet Res. 2026 Apr 29;28:e89850. doi: 10.2196/89850 (PMC13128066; doi:10.2196/89850)
Supplement: Multimedia Appendix 2 [file jmir-v28-e89850-s002.docx]

Table S2 Recall@k Values for Different Retrieval Window k

| **Retrieval Window k** | **Recall@k Value** |
| --- | --- |
| 1 | 0.9062 |
| 3 | 0.9162 |
| 5 | 0.9253 |
| 10 | 0.9291 |
