# Supplementary material for: Automated Identification of Nursing Diagnoses and Interventions From Nursing Records Using a Retrieval-Augmented Large Language Model Approach: Quantitative Study
Source: J Med Internet Res. 2026 Apr 29;28:e89850. doi: 10.2196/89850 (PMC13128066; doi:10.2196/89850)
Supplement: Multimedia Appendix 3 [file jmir-v28-e89850-s003.docx]

Table S3 Performance of LLMs under No RAG + Few-shot prompting for nursing diagnosis and intervention tasks

| Task | Model | Prompting Strategy | Precision | Recall | F1-score | IoU |
| --- | --- | --- | --- | --- | --- | --- |
| Nursing Diagnosis | Mistral-7B | No RAG + Few-shot | 0.2848 | 0.2449 | 0.2550 | 0.2439 |
|  | Qwen3-14B | No RAG + Few-shot | 0.2785 | 0.2470 | 0.2553 | 0.2470 |
|  | Llama3.3-70B | No RAG + Few-shot | 0.2848 | 0.2492 | 0.2584 | 0.2492 |
|  | Deepseek-R1 | No RAG + Few-shot | 0.2996 | 0.2733 | 0.2767 | 0.2646 |
| Nursing Intervention | Mistral-7B | No RAG + Few-shot | 0.2717 | 0.1604 | 0.1923 | 0.1543 |
|  | Qwen3-14B | No RAG + Few-shot | 0.2916 | 0.2605 | 0.2698 | 0.2451 |
|  | Llama3.3-70B | No RAG + Few-shot | 0.2289 | 0.2009 | 0.2088 | 0.1968 |
|  | Deepseek-R1 | No RAG + Few-shot | 0.4561 | 0.3511 | 0.3783 | 0.3156 |
